# Time to Deliver on Promises: The Role of ERBB2 Alterations as Treatment Options for Colorectal Cancer Patients in the Era of Precision Oncology

**DOI:** 10.3390/jpm13121701

**Published:** 2023-12-12

**Authors:** Soeren M. Buchholz, Nelia Nause, Ute König, Johanna Reinecke, Benjamin Steuber, Christoph Ammer-Herrmenau, Kirsten Reuter-Jessen, Hanibal Bohnenberger, Lorenz Biggemann, Friederike Braulke, Albrecht Neesse, Volker Ellenrieder, Philipp Ströbel, Marius Adler, Alexander König

**Affiliations:** 1 Department of Gastroenterology, Gastrointestinal Oncology and Endocrinology, University Medical Center Göttingen, 37075 Göttingen, Germany; 2 Göttingen Comprehensive Cancer Center (G-CCC), University Medical Center Göttingen, 37075 Göttingen, Germany; 3 Institute of Pathology, University Medical Center Göttingen, 37075 Göttingen, Germany; 4 Institute of Diagnostic and Interventional Radiology, University Medical Center Göttingen, 37075 Göttingen, Germany; 5 Department of Gastroenterology, University Hospital Augsburg, 86156 Augsburg, Germany

**Keywords:** HER2, ERBB2, colorectal cancer, molecular tumor board, genomic profiling, targeted therapy

## Abstract

*Receptor tyrosine kinase erythroblastic oncogene B2* (*ERBB2*), also known as *human epidermal growth factor receptor 2* (*HER2*), represents an oncogenic driver and has been effectively targeted in breast and gastric cancer. Recently, next-generation sequencing (NGS) discovered *ERBB2* as a promising therapeutic target in metastatic colorectal cancer (mCRC), where it is altered in 3–5% of patients, but no therapies are currently approved for this use. Herein, we present the experience of a single center in diagnosing actionable genetic *ERBB2* alterations using NGS and utilizing the latest therapeutic options. Between October 2019 and December 2022, a total of 107 patients with advanced CRC underwent molecular analysis, revealing actionable *ERBB2* mutations in two patients and *ERBB2* amplifications in two other patients. These findings correlated with immunohistochemical (IHC) staining. Of these four patients, two were treated with trastuzumab-deruxtecan (T-DXd). We present two exemplary cases of patients with actionable *ERBB2* alterations to demonstrate the effectiveness of T-DXd in heavily pretreated *ERBB2*-positive mCRC patients and the need for early molecular profiling. To fully exploit the potential of this promising treatment, earlier molecular profiling and the initiation of targeted therapies are essential.

## 1. Introduction

Colorectal cancer (CRC) is the third most diagnosed cancer, and despite the great scientific advances of the last 20 years, it is the second biggest cause of cancer-related death [1]. The combination of established cytotoxic chemotherapy regimens with monoclonal antibodies (mAb) depending on rat sarcoma (RAS) and B-Raf proto-oncogene, serine/threonine kinase (BRAF) mutational status as standard of care led to a median overall survival (OS) of approximately 36 months for metastatic CRC (mCRC) [2]. Recently approved treatments targeting rare conditions, including microsatellite instability (MSI), lead to a further prolonged OS in affected patients [3,4].

Identification of genetic alterations in the *erythroblastic oncogene B2* (*ERBB2*) gene (formerly referred to as *human epidermal growth factor receptor 2* (*HER2*)) such as amplifications or activating mutations revealed a new oncogenic driver alteration in 3–5% of mCRC patients with potential target implications [5,6]. The *ERBB2* gene, located on chromosome 17q21, encodes for a transmembrane glycoprotein receptor that has tyrosine kinase activity, belongs to a family of epithelial growth receptors, and activates various downstream signal transduction pathways [7]. *ERBB2* oncogene amplification or protein overexpression results in excessive mitogenic signaling, leading to uncontrolled cell growth and tumorigenesis. Additionally, *ERBB2* mutations in the extracellular, transmembrane, or cytoplasmic domains can also activate proliferation signals similarly to amplification [7,8]. Established scoring systems for the evaluation of *ERBB2* status in CRC, like the HERACLES criteria, rely on immunohistochemical stains (IHC) for quantifying expression and fluorescence in situ hybridization (FISH) for quantifying gene amplification [9]. While these diagnostic procedures are standard of care in breast cancer and gastric cancer, where ERBB2 is a more frequent target, mCRC patients are not regularly screened for *ERBB2* alterations in most centers [10].

In mCRC, comprehensive genomic profiling (CGP) using NGS is being increasingly adopted for the identification of multiple genomic changes, such as single-nucleotide variants and copy number changes [11]. Notably, copy number changes for *ERBB2* have been shown to strongly correlate with overexpression levels detected by IHC, and actionable *ERBB2* mutations are now commonly included in NGS panels for CGP of gastrointestinal tumors [12,13]. As a result, the integration of NGS into routine diagnostic workflows for advanced, therapy-resistant mCRCs at centers has made it feasible to perform broad screening for *ERBB2* mutations in this patient population for the first time.

Despite the lack of evidence supporting the use of the ERBB2-targeting mAb trastuzumab in combination with cytotoxic chemotherapy for metastatic colorectal cancer (mCRC), recent studies have indicated that alternative ERBB2-targeted therapies such as trastuzumab-deruxtecan (T-DXd), the combination of trastuzumab with the tyrosine kinase inhibitor (TKI) lapatinib, the ERBB2-targeting mAb pertuzumab, or the ERBB2 inhibitor tucatinib may offer more favorable outcomes in patients with mCRC [14,15,16,17].

In the following analysis, we analyze the role of *ERBB2* modifications in mCRC patients presented on the molecular tumor board (MTB) of the University Medical Center Göttingen from 2019–2022.

## 2. Materials and Methods

### 2.1. Molecular Tumor Board

Starting in 2019, the CGP results of patients treated at the University Medical Center Göttingen have been discussed in the interdisciplinary MTB. The MTB is part of the Comprehensive Cancer Center Niedersachsen (CCC-N), a certified tertiary cancer center. The MTB is a multidisciplinary team comprising clinicians, pathologists, tumor geneticists, and precision oncology experts who evaluate CGP results while considering the patients’ medical history and clinical situation. Based on comprehensive interdisciplinary discussions and literature research, the interdisciplinary team makes treatment recommendations for approved targeted therapies, off-label therapies, and available clinical trials, where possible. External physicians may refer patients to the MTB to discuss available CGP results. If a therapeutic recommendation is made, the team indicates the evidence level, following the European Society of Medical Oncology (ESMO) Scale for Clinical Actionability of Molecular Targets and the National Center for Tumor Diseases (NCT). Ultimately, the treating physician makes the decision to follow the recommendation.

### 2.2. Patient Population

This is a retrospective cohort study. All patients with colorectal cancer (ICD-10 diagnoses C18.x, C19.x, and C20.x) who received CGP and were discussed in the MTB of the University Medical Center Göttingen between October 2019 and December 2022 were included in the analysis. Patients with other colorectal tumors than adenocarcinomas were excluded. The study has been approved by the local ethics committee of the University Medical Center Göttingen.

### 2.3. Sequencing Assays 

Different types of CGP techniques have been used for molecular characterization. In general, DNA and RNA have been extracted from formalin-fixed paraffin-embedded tissue (FFPE) for further analysis.

With scientific advancements, various panels have been utilized for more extensive analysis. We have categorized the panels used based on their *ERBB2* analysis capacity into three groups: panels not analyzing *ERBB2* mutations or amplifications, panels evaluating *ERBB2* mutations but not amplifications, and panels that screened for both *ERBB2* mutations and amplifications. Most patients underwent testing with a QiaSeq Custom Panel, which assesses 68 genes, including *ERBB2* for mutations and is specifically focused on mutations occurring in gastrointestinal cancers but lacks the ability to screen for amplifications. However, since June 2021, the TruSight Oncology 500 (TSO500) panel has been incorporated as an additional screening tool. TSO500 is a comprehensive panel that assesses 523 cancer-related genes, inclusive of *ERBB2*, for single-nucleotide variants, multiple-nucleotide variants, and copy-number variants and assesses MSI and tumor mutational burden (TMB). It allows for simultaneous DNA and RNA analysis and can effectively detect clinically significant *ERBB2* mutations and amplifications. Large panels such as TSO500 were employed in certain cases either preemptively, given sufficient material, or as a subsequent measure if the QiaSeq Custom GI-Panel was unable to pinpoint clinically actionable targets. A detailed list of the utilized panels is provided in Supplementary Table S1.

### 2.4. Conventional ERBB2 Evaluation

Material with CGP-confirmed actionable *ERBB2* alterations (activating gene mutations or copy number variants) was quantified for immunohistochemical ERBB2 expression using a previously described protocol and assessed by experienced pathologists according to the DAKO criteria (0 to 3+) [18].

### 2.5. Statistical Analysis

To summarize patient characteristics, we employed descriptive statistics presented as frequencies (%). 

## 3. Results

### 3.1. Patient Characteristics

**Figure 1:**
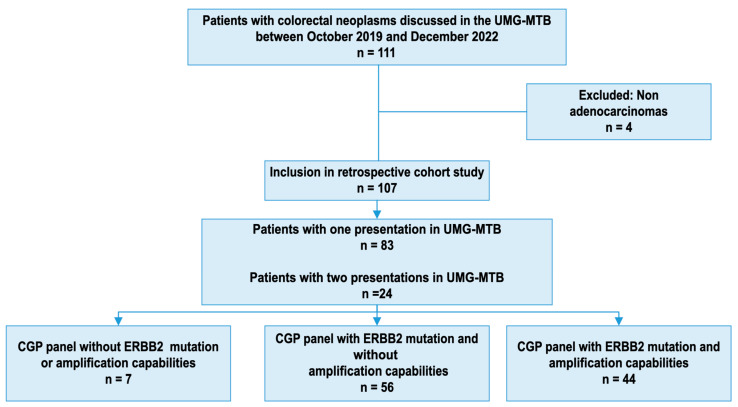
Flowchart of included patients and most comprehensive panel used for each patient for CGP. *ERBB2: Receptor tyrosine kinase erythroblastic oncogene B2*; UMG-MTB: University Medical Center Göttingen molecular tumor board; CGP: Comprehensive genomic profiling.

The CGP results of 111 patients with colorectal cancers were discussed in our MTB during the study period. Four patients with colorectal malignancies that were not adenocarcinomas were excluded; we investigated the remaining one hundred and seven patients. A total of 22% were discussed twice in the MTB based on different GCP analyses. Looking at the most comprehensive panel used in each patient, 7% of patients underwent CGP using a panel without the ability to detect *ERBB2* mutations or alterations; for 52%, a panel including *ERBB2* mutations was used; and for 41%, a panel looking at *ERBB2* mutations and amplifications was used (Figure 1). 

The median age at initial diagnosis was 54 years (range 23 to 83 years), with a population consisting of 61% men and 39% women. Most patients (50%) had tumors in the rectum, while tumors in the left (26%) and right (23%) colon were almost equally common. Most patients (61%) were initially diagnosed with metastatic cancer (UICC stage IV), but in more than one-third of the cohort analyzed, the tumor had not spread to other organs at the time of diagnosis. Baseline characteristics of the 107 investigated patients are shown in Table 1. 

**Figure 2:**
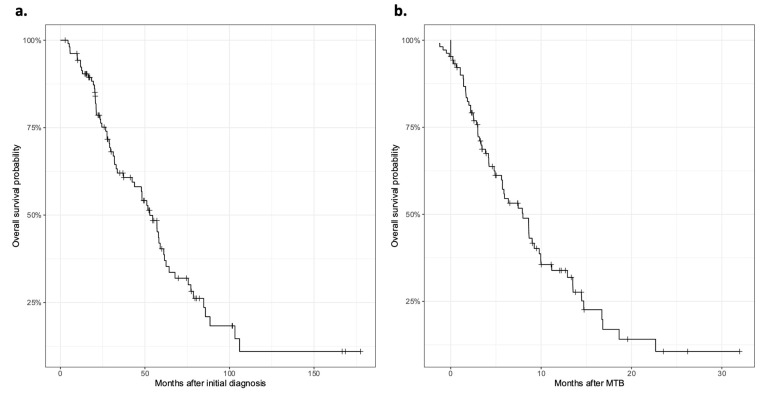
Survival of patients after initial diagnosis and after presentation on the molecular tumor board of the University Medical Center Göttingen (UMG-MTB). (**a**) The median survival of patients after initial diagnosis was 53 months; (**b**) the median survival after the first presentation in the UMG-MTB was 8 months.

Remarkably, despite a median survival period of 53 months after the initial diagnosis, the survival span following MTB presentation was only 8 months (Figure 2). The late discussion of these cases at the MTB can be attributed to the non-routine use of GCP prior to 2019, as well as the preponderance of patients undergoing advanced chemotherapy regimens without more guideline-recommended options in the MTB. Notably, in this scenario, actionable *ERBB2* alterations tend to be addressed significantly later than instances of MSI or high TMB, which are routinely evaluated through IHC following an initial mCRC diagnosis.

### 3.2. Detection and Management of CRC Patients with ERBB2 Amplification or Mutation

ERBB2 tissue overexpression in IHC is commonly associated with gene amplification and is routinely diagnosed in breast and gastroesophageal cancers. However, the recent identification of activating *ERBB2* mutations and their straightforward diagnosis via CGP are gaining prominence.

In the 99 patients who received CGP testing that included *ERBB2* mutation analysis, we found *ERBB2* mutations in four patients (4%). Two of these patients harbored previously defined, actionable pathogen mutations; one harbored a genetic variant of uncertain significance (VUS); and one harbored a benign mutation. The specific mutations are described in Supplementary Table S2. For the malignancy classification, we used the ClinVar database and the list of eligible *ERBB2* mutations used by Li and colleagues in the publication, which led to the initial approval for T-Dxd in NSCLC [19,20].

From the forty-six patients analyzed with a panel including *ERBB2* amplification detection, two patients (4.3%) harbored *ERBB2* amplifications. The characteristics of patients with an actionable *ERBB2* mutation or amplification are shown in Table 2. 

Screening for *ERBB2* amplification by IHC or FISH was not routinely performed, in part because, unlike breast and gastroesophageal cancers, there is no approved first-line therapy for *ERBB2*-positive mCRC patients. To investigate the relationship between the increase in copy numbers and higher expression, samples of patients with detected amplifications at the gene level in CGP were stained. As expected, both patients with *ERBB2* amplifications in CGP showed HER2 3+ expression.

### 3.3. Exemplatory Case Reviews of Patients with ERBB2 Amplification in mCRC

To illustrate the opportunities and challenges of targeted therapy for *ERBB2*-positive mCRC, we present two cases from our MTB. 

First, we present the case of an elderly male patient who was diagnosed with synchronous liver metastatic rectal adenocarcinoma in the summer of 2020. The primary tumor was non-stenotic and located 5 cm ab ano. Of note, the patient’s BRAF and RAS genes were wild type. Initial treatment started with a palliative first-line systemic regimen of FOLFOX (5-fluorouracil, folinic acid, and oxaliplatin) and the epidermal growth factor receptor (EGF-R)-targeting mAb panitumumab. However, a computed tomography (CT) scan after three months of treatment showed progression of liver metastases, although the primary tumor remained stable. 

The patient was switched to second-line therapy with 75% FOLFIRI (5-fluorouracil, folinic acid, and irinotecan) and bevacizumab for 3 months. Treatment was continued for an additional 3 months despite the mild enlargement of a single liver metastasis due to decreasing carcinoembryonic antigen (CEA) levels and an otherwise stable disease. An interim staging CT showed disease progression with the growth of liver metastases and increased CEA levels. As a result, the patient was started on a further palliative course of FOLFIRI and the vascular endothelial growth factor (VEGF)-inhibitor aflibercept, and a liver biopsy was initiated to obtain material for CGP, which was performed afterwards. The patient received four cycles of this regimen, after which his general condition made further tumor therapy impossible. In the late summer of 2021, he was admitted to an emergency hospital due to a deteriorating general condition and dyspnea. He was diagnosed with severe hyponatremia, pulmonary embolism with right heart strain and elevated infectious parameters, and bilirubinemia, the latter probably caused by cholangitis. CT scans showed extensive liver metastases, new pulmonary metastases, and compression of the vena cava, while the primary tumor remained unchanged. Regrettably, the patient passed away, succumbing to the cancer and its complications. The liver biopsy was posthumously analyzed using CGP. Remarkably, this analysis revealed an *ERBB2*-activating mutation. Although the patient was no longer alive, the formal MTB treatment recommendation based on this finding was T-DXd.

The second patient presented from our MTB is an elderly woman who was diagnosed with invasive rectal adenocarcinoma, hepatic and pulmonary metastases, and a metastatic ovarian mass in summer 2021. Following initial management, including a laparoscopic double-barreled descendostomy, she was started on first-line palliative systemic chemotherapy with FOLFIRI and panitumumab. After initial progression under this therapy, it was escalated to FOLFOXIRI + panitumumab, with oxaliplatin added. A significant response led to a bisegmentectomy of the metastasized liver segments II and III 12 months later. Unfortunately, the tumor progressed again during bridging chemotherapy.

Due to oxaliplatin intolerance that developed over time, the patient started receiving FOLFIRI + cisplatin + panitumumab again. However, this therapy had to be discontinued because of a persistent intolerance to chemotherapy. Four weeks later, the patient presented to the emergency department with brachiofacial paresis, and a right frontal metastasis was found and surgically removed. An epileptic seizure after the surgery 2 months later led to suspicion of carcinomatous meningitis based on cranial magnet resonance imaging. 

A CGP using a NGS panel tailored for gastroenterological malignancies was performed in July 2022, but no actionable mutation was found. Hence, a repeat analysis using a larger TSO500 panel, including *ERBB2* amplifications, was performed. This revealed a 19.4-fold amplified *ERBB2* gene, leading to an application for off-label therapy with T-DXd (intravenous application of 6.4 mg/kg bodyweight every three weeks), as per the DESTINY-CRC-01 trial.

**Figure 3:**
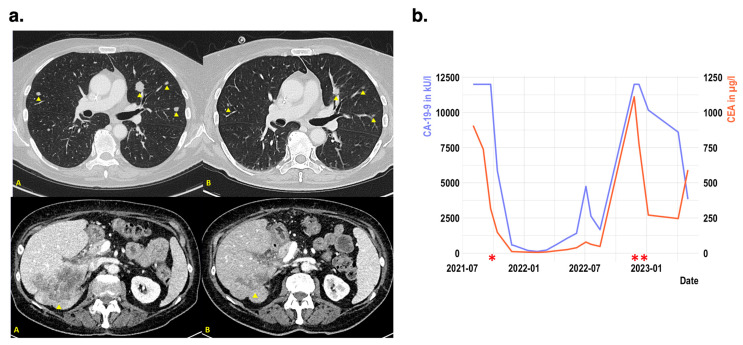
Clinical response to T-DXd in a patient with *Receptor tyrosine kinase erythroblastic oncogene B2* (*ERBB2*) amplified rectal cancer (**a**): Figure 3a Initial computed tomography scans of the liver and lungs before treatment (A) and after four cycles of trastuzumab-deruxtecan (T-DXd) in early 2023 (B). The metastases (indicated by yellow arrows) in the lung and liver show significant regression (**b**): Development of serum tumor markers carbohydrate antigen (CA) 19-9 and carcinoembryonic antigen (CEA), beginning with initial diagnosis. * Indicates the initiation of chemotherapy + panitumumab, ** indicates the initiation of T-DXd-treatment. Measurement of CA19-9 is limited to 12,000 kU/L because of technical limitations.

This therapy was initiated at the end of 2022, after the patient’s clinical recovery. After three months of therapy, a CT staging revealed a significant objective tumor response with size-reducing pulmonary and hepatic metastases (partial remission; Figure 3a). Serum tumor markers carbohydrate antigen (CA) 19-9 and CEA were also significantly reduced (Figure 3b). The objective tumor response was accompanied by a significant increase in quality of life and an increased performance status from ECOG 2 to 0. In parallel, the patient did not suffer from any severe therapy-related complications.

## 4. Discussion

Our study demonstrates that molecular profiling with NGS can uncover actionable *ERBB2* alterations and amplifications in mCRC patients. We identified *ERBB2* alterations in 4% of patients, in line with previous research suggesting an occurrence of 3–5% in mCRC [5,6,21,22]. Furthermore, we observed a promising response in a patient treated with T-DXd, an ERBB2-targeted therapy, underscoring the value of targeted therapies in improving patient outcomes.

A critical issue we encountered in this study was the late presentation of mCRC patients to the MTB. The late arrival of cases can be attributed to the non-routine use of comprehensive genomic profiling in the CGP prior to 2019 and the preponderance of patients undergoing advanced chemotherapy regimens without more guideline-recommended options. Therefore, implementing earlier molecular profiling could significantly impact the management of these patients, enabling the initiation of targeted therapies in a timelier manner. Hence, promoting routine CGP immediately following the initial diagnosis of mCRC may encounter financial obstacles due to limitations in insurance coverage. This might also drive preference towards treatments sanctioned by established guidelines, as they are more likely to be covered by insurance in many countries, despite the potential advantages that personalized therapies enabled by CGP might offer [23,24,25,26]. Although a compact NGS panel, which tests for key alterations, has become standard of care in certain entities, such as non-resectable non-small cell lung cancer (NSCLC), initial molecular testing in mCRC is typically restricted to RAS, BRAF, and MSI status, despite some recently released guidelines recommending *ERBB2* testing [27,28]. Interestingly, a cost analysis for NSCLC found that the expenses associated with testing alterations in nine relevant genes through NGS-based CGP were lower compared to conventional sequential PCR tests [29].

As recent studies in HER2low (HER2 score 1+ or 2+) breast cancer suggest, combined detection of *ERBB2* by FISH and NGS can be used for subtyping tumors with *ERBB2* alterations or amplifications [30,31]. Although there are no data on the direct clinical impact of this association, tumors with an inconclusive amplification copy number of *ERBB2* may represent an interesting target for novel ERBB2-targeted therapies. These patients could be found earlier using an approach of initial FISH for *ERBB2,* followed by early NGS in cases of (even low) *ERBB2* expression, and conversely, patients with inconclusive *ERBB2* copy numbers on NGS analysis could benefit from subsequent FISH analysis. As these studies focus on breast cancer, further investigation of the mutational landscape of HER2low mCRCs with a focus on upstream regulators of ERBB2 is needed.

As T-DXd is not approved by the US Food and Drug Administration (FDA) or the European Medicines Agency (EMA) for the treatment of mCRC yet, the patients described here were treated off-label. If the promising results of the DESTINY-CRC01 trial are confirmed, approval may be possible following the results of the phase 2 DESTINY-CRC02 trial [14,32]. The combination of tucatinib and trastuzumab for mCRC was recently approved by the FDA. While the good initial response to ERBB2-targeted therapies in mCRC faded in some cases after a few months, a sequence of different ERBB2-targeted regimens and conventional chemotherapy could lead to prolonged overall survival in *ERBB2*-positive mCRC patients.

While this study predominantly focuses on mCRC, adjuvant or neoadjuvant treatments with trastuzumab alone or in combination with pertuzumab are established for surgically resectable breast cancer [33,34,35]. An ongoing clinical trial is currently evaluating the role of T-DXd as adjuvant therapy for early-stage ERBB2 low breast cancer [36]. Typically, resectable CRCs do not undergo molecular alteration analysis. However, the groundbreaking trial by Cercek and colleagues—where all MSI-positive rectal cancers treated with the anti-PD-1-mAB dostarlimab resulted in complete remission—may challenge this dogma [37]. Despite the side effects, particularly cardiovascular, associated with trastuzumab-based regimens, limited neoadjuvant or adjuvant use of T-DXd may be a more tolerable option compared to conventional platinum-based adjuvant cytotoxic chemotherapy. Consequently, conducting trials with T-DXd in this patient group could potentially contribute to the prevention of cancer recurrence and a higher quality of life.

While our results are encouraging for the early detection and treatment of patients harboring genetic alterations of the *ERBB2* gene, there are several limitations to this study, including its retrospective nature. In addition, as a single-center study, our findings may not be generalizable to other settings or populations. Another limitation of this study is that only 44 of the 107 patients were analyzed with a panel that allows detection of *ERBB2* amplification. A total of fifty-six tumors were sequenced for *ERBB2* mutations only, and seven tumors were not analyzed for *ERBB2* at all. Moving forward, it is critical to integrate more comprehensive CGP using NGS into the early routine diagnostic workflow for mCRC. Expanding the application of NGS in clinical practice could enable the early identification of actionable alterations, potentially leading to the timelier initiation of targeted therapies. 

In conclusion, our study highlights the critical role of molecular profiling in mCRC management, demonstrating the potential of ERBB2 as a target in a subset of mCRC patients and the potential benefits of ERBB2-targeted therapies. These findings emphasize the need to further exploit the potential of these promising treatments, which would necessitate earlier and more comprehensive molecular profiling and the initiation of targeted therapies.

## Figures and Tables

**Table 1:** Baseline characteristics of the included patients (n = 107). UICC: Union for International Cancer Control; MTB: Molecular tumor board; CGP: Comprehensive genomic profiling; *ERBB2: Receptor tyrosine kinase erythroblastic oncogene B2*.

Characteristic		Number of Patients
Sex		
	Male	65 (60.7%)
	Female	42 (39.3%)
Primary tumor localization		
	Caecum/appendix	10 (9.3%)
	Ascending colon	8 (7.5%)
	Transverse colon	5 (4.7%)
	Descending colon	3 (2.8%)
	Sigmoid colon	21 (19.6%)
	Rectum	59 (55.1%)
	More than one primary tumor location	1 (0.9%)
UICC stage at diagnosis		
	I	1 (0.9%)
	II	9 (8.4%)
	III	28 (26.2%)
	IV	65 (60.7%)
	Unknown	4 (3.7%)
Age at diagnosis in years		
	Median	54.3
	Range	23.4 to 83.1
Age at presentation in MTB in years		
	Median	57.3
	Range	23.8 to 83.3
Number of MTB presentations with individual GCP		
	One	83 (77.6%)
	Two	24 (22.4%)
*ERBB2* scope of most comprehensive panel used for GCP		
	No analysis of *ERBB2*	7 (7.5%)
	Detection of *ERBB2* mutations	56 (52.3%)
	Detection of *ERBB2* mutations & alterations	44 (41.1%)
Status at last follow up		
	Deceased	64 (59.8%)
	Alive	43 (40.2%)

**Table 2:** Baseline characteristics of patients with actionable *Receptor tyrosine kinase erythroblastic oncogene B2* (*ERBB2*) mutations or amplifications.

Characteristic		Number of Patients
Sex		
	Male	2 (50%)
	Female	2 (50%)
Primary tumor localisation		
	Sigmoid colon	1 (25%)
	Rectum	3(75%)
Age at diagnosis in years		
	Median	62
	Range	25–66
Received ERBB2 specific treatment		
	Yes	2 (50%)
	No	2 (50%)
Status at last follow up		
	Deceased	2 (50%)
	Alive	2 (50%)

## Data Availability

The datasets used and/or analyzed during the current study are available from the corresponding author on reasonable request. The data are not publicly available due to privacy issues.

## Supplementary Materials

The following supporting information can be downloaded at: https://www.mdpi.com/article/10.3390/jpm13121701/s1, Table S1: NGS panels used for CGP, Table S2: Specific ERBB2 mutations in analyzed samples.
